# Determinants of adverse childhood experiences and substance use among emerging adults in the context of sustainable development goals

**DOI:** 10.1371/journal.pone.0324131

**Published:** 2025-05-27

**Authors:** Ajith K. Remesan, Varalakshmi Chandra Sekaran, Teddy Andrews Jaihind Jothikaran, Kalpana Goel, Lena Ashok

**Affiliations:** 1 Department of Global Public Health Policy and Governance, Prasanna School of Public Health, Manipal Academy of Higher Education, Manipal, Karnataka, India; 2 Department of Social and Health Innovation, Prasanna School of Public Health, Manipal Academy of Higher Education, Manipal, Karnataka, India; 3 Justice & Society, University of South Australia, Whyalla Norrie, Australia; Kasturba Medical College, Manipal Academy of Higher Education (MAHE), INDIA

## Abstract

**Introduction:**

Adverse childhood experiences (ACEs) are linked to an increased risk of developing substance use among emerging adults, which adversely affects achieving the Sustainable Development Goals (SDGs). This study explored various sociocultural and socio-economic factors associated with emerging adults as predictors of ACEs and substance use.

**Methods:**

Data was collected from 957 emerging adults studying bachelor’s degrees in 12 colleges at Mangalore University in the Udupi district, Karnataka, India. The tools for data collection included sociodemographic pro forma, Alcohol Smoking and Substance Involvement Screening tool developed by the World Health Organization (WHO ASSIST version 3.0) to assess substance use, and the Adverse Childhood Experiences Scale to determine the adverse experiences in childhood.

**Results:**

The study found that the prevalence of substance use was 17.97%, while the prevalence of Adverse Childhood Experiences (ACEs) was 18.91% in the sample examined (172 and 181 participants among the total sample of 957, respectively). The mean age for substance use initiation was 17.26 years. ACEs operated as a risk element for emerging adults’ substance use, whereas living with parents protected against it. In addition, the parents who lived together and served as primary caregivers could protect against ACEs. Family and peer group substance use was found to be associated with an increased likelihood of ACEs.

**Conclusion:**

Various sociocultural and socioeconomic factors predicted substance use and ACEs. The significance of family was revealed in the study since families without substance use and parental supervision served as a defense against both substance use and ACEs.

## 1. Introduction

Substance use and adverse childhood experiences (ACEs) are two risk factors that increase the burden of mental health concerns globally across various populations, including emerging adults. ACEs refer to childhood exposure to domestic violence, experiences of various types of abuse, neglect, and significant domestic problems, including substance misuse. ACEs also include broader societal issues such as poverty, malnutrition, peer pressure, and acts of violence in the community [1]. Emerging adulthood, which spans the ages of 18–25 years [2], is a transitional stage between adolescence and adulthood, and are particularly vulnerable to alcohol use and other substances use because of their unique needs to adapt to a new social position and the uncertainties that go along with it [3]. The use of alcohol, tobacco and other legal and illegal substances is significantly more prevalent among emerging adults with a history of ACEs such as sexual abuse, household dysfunctions including family substance use and emotional and physical abuse as well as neglect [4–7].

The Sustainable Development Goals (SDGs) target 3.5 focuses on preventing and treating substance use, including narcotic drug abuse worldwide under the goal three, good health and wellbeing [8]. There was a rise in substance use during and after the pandemic, and it was one of the factors which had an adverse effect on achieving almost all the SDGs [9,10]. A recent report from the USA found emerging adults have the highest prevalence of alcohol and illegal substance use [11]. The World Health Organization (WHO) points out that substance use during emerging adulthood has been associated with exposure to ACEs. ACEs are among the major and common reasons for stresses that individuals may encounter [1,12]. The United Nations listed ACEs as one of the top worldwide challenges for achieving the SDGs [13]. According to WHO, between the ages of 2 and 17, one billion children are subject to physical, emotional, or sexual abuse annually, highlighting the dangerously high burden of childhood trauma worldwide [14]. Developing and implementing beneficial interventions, as well as broadening the awareness of childhood trauma globally, are essential components of achieving Goal 16.2 of the 2030 SDG, which addresses eradicating violence against children [15].

The global literature on the prevalence of ACEs reveals, the overall prevalence of ACEs among the young people in Russia was 84.6%, and 28.2% of respondents had faced at least two ACEs, whereas 17.5% had been affected by four or more ACEs [16]. The study conducted in Singapore among 1130 youth found that ACEs were continuously connected to substance use in emerging adults [6]. The meta-analyses conducted across 102 studies, (n = 901,864), found that 42.32% of participants were male, reported a significant association between ACEs and smoking, problematic alcohol use, cannabis use and illicit drug use [17]. In USA 50.2% of emerging adults reported alcohol use, and 29.5% reported binge drinking in 2022 [18]. In addition, studies indicate that emerging adults who have faced at least one type of ACEs are more likely to get addicted to substances in comparison with the population without experiencing ACEs [19]. A polycentric cross-sectional research carried out in 15 different states of India, involving 1,630 youths who were visiting primary health centers reported a substance use prevalence of 32.8% with a median substance use initiation age of 18 years, with tobacco (26.4%), alcohol (26.1%) and cannabis (9.5%) mentioned as the major substances used [20]. The 2018 study conducted in the state of Kerala in India found that 91% of young people had encountered at least one ACE, while 50% had faced three or more ACEs. The study also reported that gender has a significant impact on the intensity of ACEs, with men being more likely than women to undergo psychological, physical, and sexual abuse [21]. Thus, the literature shows emerging adults have experienced various ACEs.

In addition, emerging adults are predisposed to substance use due to the existence of risk factors and a lack of protective factors [22]. Studies point out that social support, a positive family environment and community solidarity are socio-cultural factors that protect different populations against substance use [23]. Research has shown that not having these sociocultural aspects might increase the likelihood of substance use initiation [24]. Pressure from peer groups, inadequately equipped educational institutions, poverty, lack of parental supervision and interactions, household dysfunctions, and easy availability of substances are highlighted to be some of the risk factors leading to increased substance use [25].

This study aimed at investigating the prevalence and predictors of ACEs and substance use among emerging adults in Udupi district of Karnataka state in India due to the lack of data on the association between ACEs and substance use in the emerging adult population in this region.

## 2. Materials and methods

### 2.1. Study setting and design (Fig 1)

To establish an epidemiological baseline, a cross-sectional study, in the undergraduate colleges affiliated with Mangalore University were the primary units of sampling and the study was carried out in the Udupi district, Karnataka, India. Data was collected between June 2023 and October 2023 among undergraduate emerging adults. The study employed a stratified sampling method, categorizing colleges into three types: government, aided, and private. Information was gathered from the university’s website to compile a list of 10 aided colleges, 12 government colleges and 12 private colleges within the district and shortlisted five aided colleges, five government colleges, and two private colleges through simple random sampling based on the number of streams they provided and the proportion of students. In determining the sample size of students for the study, the initially calculated sample size was 385. However, given the anticipated population of students being 18,000, the study incorporated the finite population correction to adjust the sample size. The adjusted sample size was calculated considering a predicted 20% drop-out rate and a design effect of two. This correction yielded a final sample size of 944, ensuring that the study maintains its statistical power and relevance despite the anticipated challenges. Upon approval from the respective colleges, data was collected across different streams of education, such as arts, commerce and science, treating each classroom as a cluster. The average number of emerging adults per classroom ranged from 60 to 70 and data were collected from 957 emerging adults as it was following a cluster sampling.

**Fig 1:**
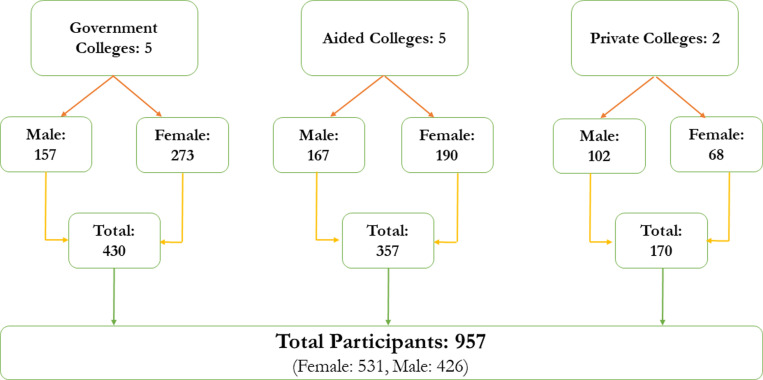
Sample distribution.

### 2.2. Approvals

Ethics approval (IEC1: 378/2022) was obtained from Kasturba Medical College and Kasturba Hospital Institutional Ethics Committee, Manipal, Karnataka, India’s institutional ethics committee. Permission from the Registrar of Mangalore University was obtained to conduct the study at the colleges in the Udupi district and permission was obtained from the heads of all colleges where the data collection took place. Written informed consent was obtained from all student participants before data collection.

### 2.3. Data collection instruments

The data collection tools consisted of a socio-demographic pro-forma, the WHO ASSIST version 3.0 [26], and the Adverse Childhood Experiences Scale (ACE) [27]. The sociodemographic pro-forma recorded the relevant characteristics of the participants, such as age, gender, living arrangement, type of family, economic status, primary caregiver, and marital status of parents. The WHO ASSIST tool focused on substance involvement among emerging adults. The substances included alcohol, tobacco, cannabis, amphetamine-type stimulants, cocaine, inhalants, hallucinogens, sedatives or sleeping pills, opioids and others. These domains helped us to understand the substance use prevalence, and the total score of each domain guided in understanding the emerging adults at moderate risk and high risk of health and other issues such as social, legal, financial, and relationship due to the participants’ pattern of substance use. Higher scores for each substance indicated a higher risk. For alcohol, scores from 0 to 10 represented a low-risk group, 11–26 represented a moderate-risk group and scores from 27 and more represented a high-risk group. For all the other substances, scores from 0 to 3 were considered low risk, 4–26 were considered moderate risk, and 27 and above were considered high-risk groups.

The ACE questionnaire has ten questions, each answered by selecting yes or no. The first three questions refer to emotional, physical and sexual abuse, respectively. Questions 4 and 5 assess emotional and physical neglect, and 6–10 refer to household dysfunctions of the participants. Data was collected using both English and local language (Kannada) survey forms. The translated survey form was reverse-translated to ensure the accuracy of the instrument. The overall internal consistency (Cronbach α) for the ACE questionnaire was 0.654.

### 2.4. Data collection procedure

To maximize participant convenience, the data collection was carried out in a classroom setting. Written informed consent was acquired before data collection, and a participant information sheet was shared with each participant. Confidentiality was sought in the completion of the self-administered surveys by the participants.

### 2.5. Data analysis

Descriptive analysis and stepwise backward logistic regression were performed using SPSS version 25 (IBM, Bangalore, India) to identify predictors. We computed descriptive statistics for each variable, followed by regression analysis. Proportions were compared using Chi-square and Fisher’s exact test, with significance at p < 0.05 to assess the association between the variables.

## 3. Results

### 3.1. Sociodemographic details of the participants (Table 1)

Among the 957 participants, 531 were female, and 426 were male. Most of them were 18–20 years old (85.79%), and the participants’ mean age was 19.43 ± 1.06 years. Among the participants, 50.47% were in their first year of college, and 56.63% were studying the commerce stream. Almost three-quarters of the participants were from a nuclear family (74.19%), and 76.38% belonged to below the poverty line. Majority of the participants (96.86%) had their parents as primary caregivers in early years (0–5 years), and 80.67% lived with their parents currently. Moreover, 92.16% of the participant’s parents were living together. Most participants had one or more siblings (87.88%), and more than half were their family’s firstborn child (53.08%).

**Table 1:** Sociodemographic Characteristics of Participants (n = 957).

Sociodemographic variables		Frequency (%)
Age	18-20 years	821 (85.79)
	21-25 years	136 (14.21)
Gender	Female	531 (55.49)
	Male	426 (44.51)
Year of study	First year	483 (50.47)
	Second year	338 (35.32)
	Third year	136 (14.21)
Type of educational institution	Aided	357 (37.3)
	Government	430 (44.93)
	Private	170 (17.77)
Stream of course	Arts	62 (6.48)
	Commerce	542 (56.63)
	Science	353 (36.89)
Living arrangement	Living with parents	772 (80.67)
	Others	185 (19.33)
Type of family	Nuclear	710 (74.19)
	Joint/Extended	247 (25.81)
Economic status	Above poverty line	226 (23.61)
	Below poverty line	731 (76.39)
Believe in religion	Yes	922 (96.34)
	No	35 (3.66)
Religion	Hindu	868 (90.7)
	Others	89 (9.3)
Number of siblings	No siblings	116 (12.12)
	One or more siblings	841 (87.88)
Birth order	First born	508 (53.08)
	Second born/ other	449 (46.92)
Primary caregiver (0–5 years)	Parents	927 (96.86)
	Others	30 (3.14)
Marital status of parents	Parents living together	882 (92.16)
	Divorced/ Separated/ Widowed	75 (7.84)

### 3.2. Prevalence of substance use and ACEs (Table 2)

The substance use prevalence was 17.97%, and the mean age of first use was 17.26 years. The sources of initial substance use included family (29.65%), peers (19.77%), and self (26.74%). The majority of the participants (73.56%) mentioned their friends use substances; for 13.58% of participants, at least one of the family members was using substances, and 4.39% mentioned that their siblings were using substances. Adverse childhood experiences were faced by 18.91% of the participants while growing up. Among the participants who reported ACEs, 8.6% experienced emotional abuse; 7.5% reported physical abuse, 2.2% were sexually abused; 5.1% experienced emotional neglect, 5.1% faced physical neglect, 1.3% and 9.72% experienced household dysfunction during their childhood.

**Table 2:** Substance use and ACEs among the participants (n = 957).

Number of substances used	Frequency (%)
None	785 (82.02)
Only One	105 (10.97)
Two	41 (4.28)
Three or More	26 (2.71)
**Adverse childhood experiences**	**Frequency (%)**
Yes	181 (18.91)
No	776 (81.1)

*Note: Substances included alcohol 136 (14.2%), tobacco 39 (4.1%), cannabis 15 (1.6%), amphetamine-type stimulants 4 (0.4%), cocaine 25 (2.6%), inhalants 41 (4.3%), sedatives or sleeping pills 7 (0.7%) and hallucinogens 8 (0.8%).*

*ACEs: Emotional abuse 82 (8.6%), physical abuse 72 (7.5%), sexual abuse 21 (2.2%), emotional neglect 49 (5.1%), physical neglect 12 (1.1%) and household dysfunction 93 (9.72%).*

### 3.3. Association between sociodemographic variables with substance use and ACEs

Substance use among emerging adults showed a highly significant association with socio-demographic variables such as the age of the participant, gender, year in which they are studying, type of educational institute, stream of their course, living arrangement, economic status of the family, age and source of substance use initiation, and substance use by siblings, family members and peer groups (p < 0.001). The association was significant for the type of their family and the number of siblings they had (p < 0.05). ACEs showed a highly significant association with the age of the participants, the year in which they are studying, the stream of their course, their living arrangement, age of substance use initiation, source of substance use initiation and substance use by siblings, family members and peer groups (p < 0.001). In addition, the association between ACEs and primary caregivers during the early years was significant for the participants (p < 0.05).

### 3.4. Predictors of substance use among participants (Table 3)

Backward logistic regression was performed to find the predictors of the outcome variables ACEs and substance use. Univariate and multivariate analyses for predictors of substance use were conducted. Factors such as age, gender, year of study, type of educational institute, stream of course, living arrangement, type of family, economic status, mother’s occupation, number of siblings, marital status of parents, siblings’ substance use, substance use by family members, peer substance use, and ACEs were adjusted in the multivariate analysis after being found significant in the univariate analysis. Of them, age, type of educational institution, living arrangement, substance use by family members, peer substance use and ACEs showed significance in multivariate analysis and crude odds ratio (COR), adjusted odds ratio (AOR) and confidence interval (CI) were reported.

**Table 3:** Univariate and multivariate analysis for predictors of any substance use among participants.

Socio-demographic variables		Substance use						
		Yes (%)	No (%)	n (100%)	COR (95% CI),	p-value	AOR (95% CI)	p-value
Age	18-20 years	113 (13.8)	708 (86.2)	821	0.208 (0.141,0.309)	< 0.001	**0.424 (0.255,0.703)**	0.001*
	21-25 years	59 (43.4)	77 (56.6)	136	Ref	1	Ref	1
Gender	Female	65 (12.2)	466 (87.8)	531	0.416 (0.296,0.584)	< 0.001	0.653 (0.421,1.013)	0.057
	Male	107 (25.1)	319 (74.9)	426	Ref	1	Ref	1
Year of study	First year	53 (11)	430 (89)	483	0.368 (0.258,0.523)	< 0.001	0.697 (0.432,1.124)	0.138
	Second/ Third year	119 (25.1)	355 (74.9)	474	Ref	1	Ref	1
Type of educational institute	Aided	105 (29.4)	252 (70.6)	357	1.607 (1.039,2.485)	0.033	**1.727 (1.014,2.943)**	0.044*
	Government	32 (7.4)	398 (92.6)	430	0.310 (0.185,0.52)	< 0.001	0.573 (0.304,1.08)	0.085
	Private	35 (20.6)	135 (79.4)	170	Ref	1	Ref	1
Stream of course	Arts	5 (8.1)	57 (91.9)	62	0.281 (0.109,0.724)	0.009	0.688 (0.217,2.187)	0.526
	Commerce	83 (15.3)	459 (84.7)	542	0.579 (0.413,0.812)	0.002	0.799 (0.511,1.248)	0.323
	Science	84 (23.8)	269 (76.2)	353	Ref	1	Ref	1
Living arrangement	Living with parents	126 (16.3)	646 (83.7)	772	0.589 (0.401,0.865)	0.007	**0.518 (0.297,0.904)**	0.021*
	Others	46 (24.9)	139 (75.1)	185	Ref	1	Ref	1
Type of family	Nuclear	139 (19.6)	571 (80.4)	710	1.579 (1.047,2.381)	0.029	1.049 (0.643,1.713)	0.847
	Joint/ Extended	33 (13.4)	214 (86.6)	247	Ref	1	Ref	1
Economic status	Above poverty line	69 (30.5)	157 (69.5)	226	2.68 (1.885,3.808)	< 0.001	1.477 (0.954,2.287)	0.08
	Below poverty line	103 (14.1)	628 (85.9)	731	Ref	1	Ref	1
Mother’s occupation	Professional/ semi-professional	3 (27.3)	8 (72.7)	11	1.631 (0.428,6.217)	0.474	1.35 (0.284,6.416)	0.706
	Business/farming	7 (17.5)	33 (82.5)	40	0.922 (0.401,2.124)	0.849	0.424 (0.151,1.193)	0.104
	Skilled/ semi-skilled	7 (9.1)	70 (90.9)	77	0.435 (0.196,0.964)	0.04	0.485 (0.195,1.208)	0.12
	Home maker	155 (18.7)	674 (81.3)	829	Ref	1	Ref	1
Number of Siblings	No siblings	30 (25.9)	86 (74.1)	116	1.717 (1.092,2.701)	0.019	1.502 (0.88,2.562)	0.136
	One or more siblings	142 (16.9)	699 (83.1)	841	Ref	1	Ref	1
Marital status of parents	Parents living together	153 (17.3)	729 (82.7)	882	0.619 (0.357,1.071)	0.086	1.266 (0.552,2.903)	0.577
	Divorced/ Separated/ Widowed	19 (25.3)	56 (74.7)	75	Ref	1	Ref	1
Siblings substance use	No	151 (16.5)	764 (83.5)	915	0.198 (0.105,0.371)	< 0.001	0.883 (0.407,1.917)	0.754
	Yes	21 (50)	21 (50)	42	Ref	1	Ref	1
Substance use by family members	No	121 (14.6)	706 (85.4)	827	0.265 (0.178,0.397)	< 0.001	**0.51 (0.303,0.86)**	0.011*
	Yes	51 (39.2)	79 (60.8)	130	Ref	1	Ref	1
Peer substance use	No	66 (9.4)	638 (90.6)	704	0.143 (0.101,0.205)	< 0.001	**0.286 (0.181,0.451)**	< 0.001*
	Yes	106 (41.9)	147 (58.1)	253	Ref	1	Ref	1
Adverse childhood experiences	No	112 (14.4)	664 (85.6)	776	0.34 (0.235,0.492)	< 0.001	**0.592 (0.362,0.967)**	0.036*
	Yes	60 (33.1)	121 (66.9)	181	Ref	1	Ref	1

*Note: COR - crude odds ratio, AOR - adjusted odds ratio, CI – confidence interval*

With reference to participants aged 21–25 years, those aged 18–20 years reduced the likelihood of substance use (AOR 0.424, 95% CI (0.255, 0.703), p = 0.001). Emerging adults studying in aided colleges (AOR 1.727, 95% CI (1.014, 2.943), p = 0.044) showed a higher likelihood of using substances in comparison with those who study in private colleges. Parents acted as a preventive factor against substance use as those who lived with parents (AOR 0.518, 95% CI (0.297, 0.904), p = 0.021) showed less likelihood of using substances than those who stayed in hostel, as paying guests or with other relatives. The emerging adults having a family (AOR 0.51, 95% CI (0.303, 0.86), p = 0.011) and peer groups (AOR 0.286, 95% CI (0.181, 0.451), p = < 0.001), without using substances, were also acted as a protective factor against substance use in comparison with family and peer groups with the usage of substances. ACEs increased the probability of substance use among emerging adults, as those participants who never experienced any ACEs (AOR 0.592, 95% CI (0.362, 0.967), p = 0.036) were protected against substance use in comparison with those who faced ACEs.

### 3.5. Predictors of ACEs among participants (Table 4)

Following the analyses on substance use predictors, further univariate and multivariate analyses for predictors of ACEs were performed. The factors age, year in which they are studying, stream of the course studying, their living arrangement, type of family, occupation of mother, primary caregiver during the first five years of life, marital status of parents, substance use by siblings, family members and peer groups showed significance on univariate analysis and were adjusted in multivariate analysis. The predictors of ACEs that emerged included the stream of the course that the participants are studying, occupation of the mother, marital status of parents, primary caregivers, and substance use by siblings, family members and peer groups.

**Table 4:** Univariate and multivariate analysis for predictors of ACEs among participants.

Socio-demographic variables		Adverse childhood experiences						
		Yes (%)	No (%)	n (100%)	COR (95% CI)	p-value	AOR (95% CI)	p-value
Age	18-20 years	141 (17.2)	680 (82.8)	821	0.498 (0.330,0.751)	0.001	0.776 (0.463,1.302)	0.337
	21-25 years	40 (29.4)	96 (70.6)	136	Ref	1	Ref	1
Year of study	First year	76 (15.7)	407 (84.3)	483	0.656 (0.473,0.91)	0.012	0.802 (0.528,1.219)	0.303
	Second/ Third year	105 (22.2)	369 (77.8)	474	Ref	1	Ref	1
Stream of course	Arts	22 (35.5)	40 (64.5)	62	1.631 (0.92,2.894)	0.094	**1.939 (1.02,3.688)**	0.043*
	Commerce	70 (12.9)	472 (87.1)	542	0.440 (0.311,0.623)	< 0.001	**0.489 (0.332,0.72)**	< 0.001*
	Science	89 (25.2)	264 (74.8)	353	Ref	1	Ref	1
Living arrangement	Living with parents	131 (17)	641 (83)	772	0.552 (0.379,0.803)	0.002	0.936 (0.548,1.597)	0.807
	Others	50 (27)	135 (73)	185	Ref	1	Ref	1
Type of family	Nuclear	144 (20.3)	566 (79.7)	710	1.444 (0.973,2.142)	0.068	1.385 (0.895,2.143)	0.143
	Joint/Extended	37 (15)	210 (85)	247	Ref	1	Ref	1
Mother’s occupation	Professional/semi-professional	5 (45.5)	6 (54.5)	11	3.772 (1.136,12.523)	0.03	**4.64 (1.189,18.116)**	0.027*
	Business/farming	13 (32.5)	27 (67.5)	40	2.18 (1.099,4.323)	0.026	1.349 (0.61,2.984)	0.46
	Skilled/ semi-skilled	13 (16.9)	64 (83.1)	77	0.919 (0.494,1.713)	0.791	0.842 (0.427,1.662)	0.621
	Home maker	150 (18.1)	679 (81.9)	829	Ref	1	Ref	1
Primary caregiver (0–5 years)	Parents	170 (18.3)	757 (81.7)	927	0.388 (0.181,0.83)	0.015	**0.4 (0.177,0.903)**	0.027*
	Others	11 (36.7)	19 (63.3)	30	Ref	1	Ref	1
Marital status of parents	Parents living together	152 (17.2)	730 (82.8)	882	0.33 (0.201,0.543)	< 0.001	**0.409 (0.203,0.825)**	0.012*
	Divorced/ Separated/ Widowed	29 (38.7)	46 (61.3)	75	Ref	1	Ref	1
Siblings substance use	No	160 (17.5)	755 (82.5)	915	0.212 (0.113,0.397)	< 0.001	**0.453 (0.22,0.934)**	0.032*
	Yes	21 (50)	21 (50)	42	Ref	1	Ref	1
Substance use by family members	No	119 (14.4)	708 (85.6)	827	0.184 (0.124,0.274)	< 0.001	**0.285 (0.182,0.447)**	< 0.001*
	Yes	62 (47.7)	68 (52.3)	130	Ref	1	Ref	1
Peer substance use	No	94 (13.4)	610 (86.6)	704	0.294 (0.21,0.412)	< 0.001	**0.473 (0.314,0.714)**	< 0.001*
	Yes	87 (34.4)	166 (65.6)	253	Ref	1	Ref	1

Compared with the emerging adults studying the science stream, ACEs seem to be high in those who study the arts stream (AOR 1.939, 95% CI (1.02, 3.688), p = 0.043), and it seems to be low among the participants from commerce stream (AOR 0.489, 95% CI (0.332, 0.72), p = < 0.001). When the mothers of the participants work in a professional or semi-professional sector, there was a 4.64 times increased risk that the participants reported higher ACEs in comparison with the participants with mothers as homemakers (AOR 4.64, 95% CI (1.189, 18.116), p = 0.027). In addition, those who had their parents as primary caregivers during the initial five years of their lives reported fewer ACEs than those who had others as primary caregivers (AOR 0.4, 95% CI (0.177, 0.903), p = 0.027). Those with their parents living together faced comparatively fewer ACEs than those with parents separated/widowed/divorced (AOR 0.409, 95% CI (0.203, 0.825), p = 0.012). Those participants who had their siblings, other family members and their peer groups not using substances were protected against ACEs in comparison with those participants who had their siblings, family members and peer groups with the usage of substances [(AOR 0.453, 95% CI (0.22, 0.934), p = 0.032), (AOR 0.285, 95% CI (0.182, 0.447), p = < 0.001), (AOR 0.473, 95% CI (0.314, 0.714), p = < 0.001), respectively].

## 4. Discussion

The current study results showed the prevalence of substance use and ACEs as 17.19% and 18.97%, respectively, in a sample of 957 emerging adults. The prevalence of substance use varied across different populations regionally and globally. Substance use prevalence is around 8% to 10% in the general population, but this figure is significantly higher in medical settings [28]. For instance, the prevalence of substance use reaches about 20% in standard primary care clinics, increases to around 40% among general medical patients in hospitals, and exceeds 70% in emergency or urgent care facilities. This is because individuals with substance use disorders are also at a greater risk of developing chronic health conditions [28]. A large-scale study conducted in primary health centers across 15 states in India reported the prevalence of substance use as 32.8% [20]. Although the prevalence rate reported in the present study is less, the initiation age of substance use among emerging adults (17.26 ± 2.5 years) was found to be close to the existing literature, which showed 17.2 ± 2.7 years as the initiation age [20]. Similarly, the prevalence of ACEs in the present study was found to be 18.91% which is low compared to existing study conducted among 800 youths from different colleges and universities in Kashmir of India [29]. Studies indicate that the resilience of parents, strong social networks, understanding of parenting and child development, access to practical assistance during challenging times, and the social and emotional skills of children will act as a protective factor against ACEs [30]. In relation to this, the low prevalence of ACEs among participants may be attributed to most participants are living with both the parents, who also served as primary caregivers, which is a significant protective factor against ACEs, specifically in the Indian context. The higher prevalence of ACEs can also be a challenge for achieving SDG 16.2, which targets ending violence against children.

The present study further identified that living arrangement as a protective factor against substance use as well. Existing literature [31] also indicates that those who have both parents taking care of them have a lower probability of using substances than those who lack parental monitoring. Aligning with the current literature findings [32], our study also found that substance use by family members and peer groups of the emerging adults predicted substance use initiation. This may explain that being surrounded by individuals who use substances can motivate others also to experiment with those substances.

The findings of our study show that ACEs also acted as a risk factor in substance use initiation among emerging adults with 34.88% of participants who were using substances having had experienced at least one ACE during their childhood. Among young adult males in India, substance use was closely linked to ACEs, such as abuse and domestic violence [33]. They may develop substance use as a coping mechanism against ACEs, which eventually puts them at risk of developing various health issues and social problems [34]. In other words, positive childhood experiences seem to protect individuals from substance use as they grow into higher developmental stages. This is an important observation to be noted by the policy makers and other key stakeholders to achieve SDG 16.2.

Besides, we also found that ACEs were comparatively less among those who had their parents as primary caregivers during the first five years of their lives and when the parents lived together. This observation corroborates with the existing literature [35] that reports favorable family attitudes, good parental warmth and control can reduce the chances of ACEs. Furthermore, our study adds newer knowledge that those participants who had their mother as a homemaker also seemed to be protected against ACEs. Another study confirms that, in contrast to fathers’ unemployment, mothers’ unemployment was not associated with the risk of ACEs [36]. Moreover, research has also reported that ACEs due to family members not only have a negative association with individuals’ health during childhood but also have hostile effects on their health in adulthood [37]. ACEs also appear to be more prevalent among emerging adults pursuing arts streams than those in the science field, while individuals focusing on commerce-related studies tend to report lower instances of ACEs. These findings align with the existing knowledge that in comparison with youth without ACEs, who were more likely to study subjects other than social sciences, youth with four or more ACEs were reported to pursue social science streams [38]. In addition, studies indicate that personal life experiences influence career preferences and the choice of courses taken in higher education [39].

Our findings indicate that substance use and ACEs act as major hurdles to attaining various SDGs. Substance use causes a great deal of harm to people’s lives, the economy, society and the environment at large [40,41]. Sustainable development is complex due to the production and use of many legal and illicit substances connected to varied social problems and health concerns [42]. To enable individuals who promote and advocate for policy reforms, it is necessary to increase public awareness of these concerns so that it will improve their lives.

## 5. Conclusion

In the present study, socio-economic and cultural factors are associated with ACEs and substance use. In contrast with the literature available, the prevalence of ACEs was comparatively low in the current study. However, substance use prevalence was similar to the existing literature. The study also highlighted the importance of family as parental supervision and family without substance use functioned as a protective component against substance use and ACEs. Comprehensive preventative programs and improved mental health services for impacted families are necessary to address substance use and ACEs to achieve the SDGs. The root causes of these problems can also be addressed by fostering economic empowerment and reinforcing family support networks. A more encouraging environment for resilience and recovery may also be produced by promoting policy reform and community collaboration.

## 6. Limitations

The limitations of the research need to be accounted for as it is not without any. As the study used a cross-sectional design, the chances of bias cannot be neglected, and the data was only gathered from emerging adults studying in undergraduate colleges. Future research should address emerging adults in other fields of society, including those who are working. Participants may underreport their experiences since the study uses self-reported data on substance use and ACEs, which may result in inaccuracies due to factors including social desirability bias and recollection bias. Additionally, although the study concentrates on specific substances listed by the WHO, it is essential to remember that other locally available substances may not be covered entirely. It would be beneficial to carry out a longitudinal study that examines how these characteristics change over time and aids in establishing causal linkages between substance use and ACEs. The results make a compelling case for the creation of focused intervention initiatives aimed at preventing emerging adults’ substance use, especially among those who had ACEs. Future research may expand on this study by acknowledging these limitations and implementing the recommendations into action. This will eventually yield deeper and essential insights into emerging adults’ substance use and its underlying causes.
